# Synthesis of Electronically Modified Ru-Based Neutral 16 VE Allenylidene Olefin Metathesis Precatalysts

**DOI:** 10.3390/molecules17055177

**Published:** 2012-05-04

**Authors:** Martin Lichtenheldt, Steffen Kress, Siegfried Blechert

**Affiliations:** Technische Universität Berlin, Institut für Chemie, Sekr. C3, Straße des 17. Juni 135, Berlin 10623, Germany

**Keywords:** olefin metathesis precatalysts, neutral allenylidene, electronic modifications, ring closing metathesis

## Abstract

Electronic modifications within Ru-based olefin metathesis precatalysts have provided a number of new complexes with significant differences in reactivity profiles. So far, this aspect has not been studied for neutral 16 VE allenylidenes. The first synthesis of electronically altered complexes of this type is reported. Following the classical dehydration approach (*vide infra*) modified propargyl alcohols were transformed to the targeted allenylidene systems in the presence of PCy_3_. The catalytic performance was investigated in RCM reaction (ring closing metathesis) of benchmark substrates such as diallyltosylamide (**6**) and diethyl diallylmalonate (**7**).

## 1. Introduction

Metathesis represents one of the ground-breaking achievements in modern synthetic chemistry. This powerful tool for carbon-carbon bond formation in hands of synthetic chemists renders the possibility to cut an (strategically introduced) olefinic double bond in retrosynthetic studies of target molecules [1,2]. Significant representatives of Ru-based metathesis precatalysts are shown in Figue 1, where the benzylidene species **I** and **II** are most widely used [3,4,5,6]. Milestones in catalyst development have been the introduction of NHC ligands (*N*-heterocyclic carbenes) and the usage of chelating carbene moieties to provide highly active catalysts of improved stability (e.g., **II**, L = NHC). Beyond that, recent work has shown the dramatic influence of electronic variations within the benzylidene moiety in precatalysts of type **II** [7,8]. For instance, electron withdrawing *para*-substituents successfully improve the initiation rate by means of decreasing both the electron density on the benzylidene (increased electrophilicity) and the chelating isopropoxy moiety (decreased Lewis basicity).

**Figure 1:**
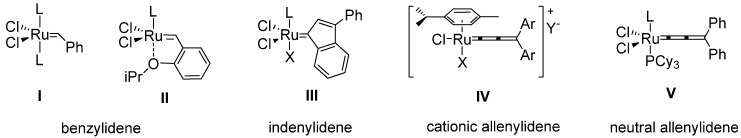
Selected examples of Ru-based olefin metathesis precatalysts; L: phosphine, NHC; X: halide; Y: counter ion.

The systematic investigation of these effects did not only reveal catalysts of enhanced activity, but moreover a fine tuning of the catalyst´s performance can generally be achieved. Furthermore, the nature of the carbene moiety is one key factor for a catalyst’s stability during metathesis, thus structural modifications additionally influence the characteristics of the dormant species and therefore the lifetime of the metathesis catalyst. Beside **I** and **II** Ru-indenylidenes and Ru-allenylidenes also became of interest. The straightforward and short synthetic approach starting from inexpensive commercially available materials combined with a remarkable thermal stability makes these precatalysts a cost-efficient and valuable complement to the well-established benzylidene systems. Electronic modifications on cationic complexes of type **IV** have been studied thoroughly [9,10,11,12], whereas in the area of indenylidenes **III**, only a few examples have been published to date [13,14]. Especially within the class of neutral 16 VE allenylidenes, two complexes of type **V** are the only examples known so far [15]. Herein, we present the first synthesis of neutral 16 VE allenylidene Ru-precatalysts bearing an electronically modified carbene moiety (compounds **5a**–**c**, Scheme 3). The reactivity profiles of these complexes were investigated during RCM of diallyltosylamide (**6**) and diethyl diallylmalonate (**7**). 

## 2. Results and Discussion

### 2.1. Concept for the Synthesis of Electronically Modified Neutral Ru-Based 16 VE Allenylidene Complexes

**Scheme 1:**
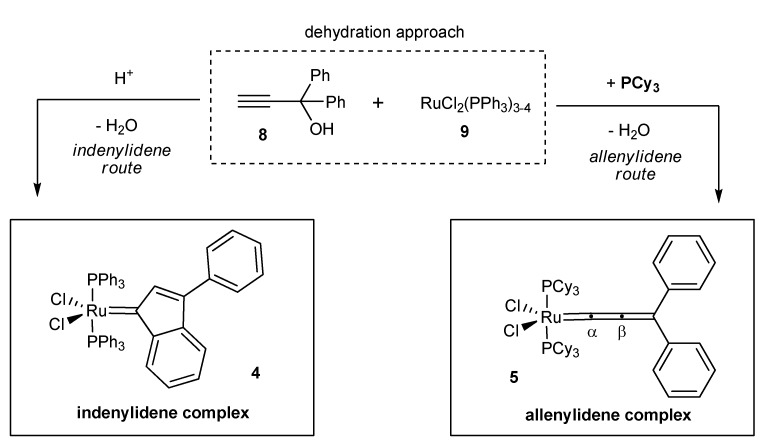
Concept for the synthesis of electronically modified neutral allenylidene and indenylidene precatalysts.

The discovery of Selegue and coworkers that Ru(II)-complexes promote the dehydration of propargyl alcohols of type **8** (Scheme 1, *dehydration approach*) marks the inception of a straightforward access to a new metathetically active Ru-based catalyst class, the cationic allenylidenes (**IV**, Figure 1) [16]. This remarkably simple route could later be extended to the synthesis of coordinatively unsaturated 16 VE species **III** and **V** by Hill [17], Nolan [15] and Fürstner [18,19]. During the first attempts of Hill to obtain neutral allenylidenes a new metathetically active complex was discovered, which would subsequently be characterized by Nolan as the corresponding Ru-indenylidene complex (type **III**, Figure 1). Investigations concerning this unexpected reaction pathway revealed, that in the presence of protons indenylidene complexes are formed by an acid catalyzed rearrangement subsequent to the formation of the respective allenylidene complexes containing PPh_3_ ligands (Scheme 1; for a detailed discussion see [20,21,22,23]). By adding PCy_3_, replacing PPh_3_ during allenylidene formation, the reaction channel to the respective indenylidene is blocked, most likely due to the increased electron density on the metal center [15]. These results offer a flexible and reliable access to both precatalyst families. 

Within these studies, propargyl alcohols with *para*-substituted aromatic moieties were chosen to access allenylidenes in the presence of PCy_3_ (*allenylidene route*, Scheme 1); substituents at this position have shown to exert the most significant electronic effect (*vide supra*).

### 2.2. Synthesis of Electronically Modified Propargyl Alcohols ***8a***–***c***

**Scheme 2:**
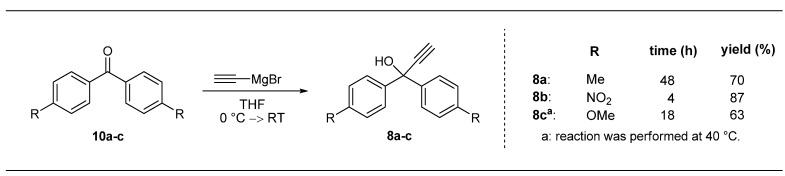
Synthesis of electronically modified propargyl alcohols **8a**–**c**.

The propargyl alcohols needed for these investigations were obtained by addition of ethynylmagnesium bromide to commercially available disubstituted benzophenone derivatives **10a**–**c** (Scheme 2). Only compound **10c** had to be synthesized by a known three step-procedure involving nitration and subsequent oxidative cleavage of triphenylmethanol [24,25,26]. The Grignard reactions afforded compounds **8a**–**c** in good isolated yields (Scheme 2); as expected by means of polarizing effects the best result was obtained for electron withdrawing NO_2_-substituted derivative (**8b**; Scheme 2). Due to the prolonged reaction time and slightly forcing conditions (**8c**, 40 °C, Scheme 2), the yield is somewhat decreased for the product containing an electron-donating OMe-substitutent.

### 2.3. Synthesis of Ru-Precatalysts ***5a***–***c***

**Scheme 3:**
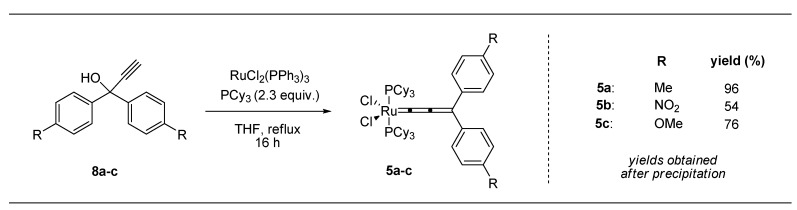
Allenylidene (compound **5a**–**c**) formation employing modified propargyl alcohols **8a**–**c**.

The electronically modified allenylidene complexes **5a**–**c** (Scheme 3) were obtained in good yields via dehydration of propargyl alcohols **8a**–**c**. In the presence of RuCl_2_(PPh_3_)_3_ (**9**) and 2.3 equiv. PCy_3_, while strictly excluding oxygen, indenylidene formation was successfully inhibited. For full conversions extended reaction times of 16 h in THF (reflux) were necessary.

**Scheme 4:**
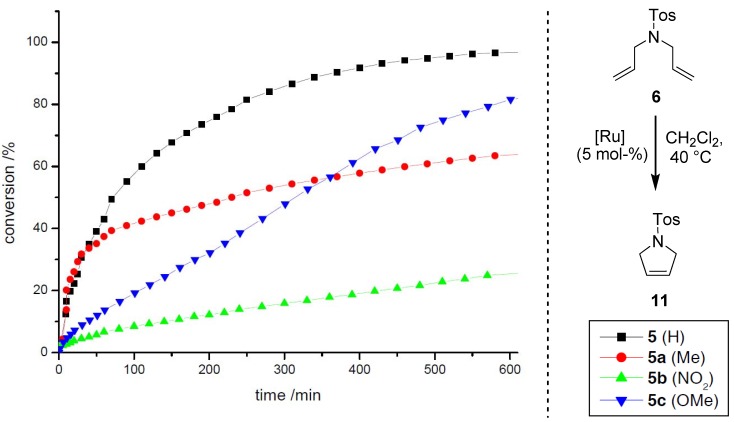
RCM of **6** employing precatalysts **5a**–**c** relative to **5**.

**Scheme 5:**
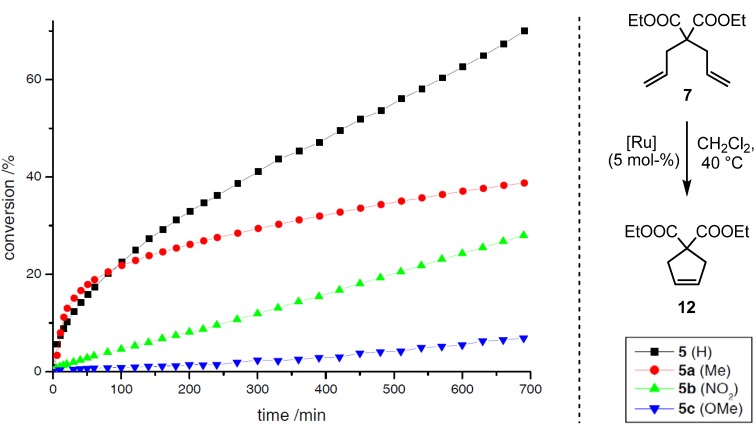
RCM of **7** employing precatalysts **5a**–**c** relative to **5**.

To separate the respective products from remaining phosphines, the crude mixtures were dissolved in a minimum amount of DCM, followed by addition of an excess of *n*-hexane to precipitate the precatalysts. The suspensions were filtrated and the solid matters were washed with cold *n*-hexane providing analytically pure compounds; the diminished yields in some cases are partly attributed to the washing procedure during the purification process. Nevertheless, this route provided the first electronically modified neutral 16 VE allenylidene complexes as intended. With this new set of precatalysts in hands we were able to investigate the influence of the modified aromatic systems on the catalytic performance during RCM of **6** and **7** (Scheme 4 and Scheme 5).

### 2.4. Reactivities of the New Precatalysts

Concerning the effects of electronic modification, disubstituted systems **5a**–**c** were evaluated during RCM of the benchmark substrates **6** and diethyl diallylmalonate (**7**); precatalyst **5** (Scheme 1) served as reference [15,27]. The reactions were performed in DCM-*d_2_* (0.12 M) with a catalyst loading of 5 mol–% at 40 °C under inert atmosphere, conversions were determined by integrating the respective olefin signals in ^1^H-NMR spectra.

The modified precatalysts show significant differences concerning their reactivity profiles during the transformation of **6** (Scheme 4) and **7** (Scheme 5). Complex **5a** (Me) initiates slightly faster than the reference system **5**, albeit a shorter lifetime is observed, presumably due to a higher concentration of active species in the early stage of the reaction. For complexes **5b** (NO_2)_ and **5c** (OMe) the conversions are significantly slower, but a high stability is observed in both cases, which is reflected in the reaction progress even after prolonged reaction times. In the course of the transformation, a conversion of 57% is observed for **5** after 100 min, whereas **5a** (42%), **5c** (20%) and **5b** (9%) catalyze the reaction less efficiently. In comparison to this, the results for the reaction of **7** show similar trends. For both complexes **5** and **5a** a conversion of 20% is determined after the same time, here again the Me-derivative **5a** initiates considerably faster. Precatalysts **5b** and **5c** catalyze the RCM inefficiently, whereas in contrast to the former reaction nitro-derivative **5b** shows a higher activity than **5c**. In contrast to the studies on electronic modifications within precatalyst of type **II** (*vide supra*), no correlation with regard to the electron density at the aromatic system can be found at the first glance. Nevertheless, the enhanced initiation rate for the Me-derivative in comparison to the acceptor and donor substituted complexes **5b** and **5c**, respectively, is remarkable; further investigations are required to get a deeper insight into the important coherencies influencing the stability *and* reactivity of **5a** relative to **5b** and **5c**. These first results clearly show, that modifications within the aromatic backbone of the allenylidene open access to new metathetically active neutral 16 VE complexes with considerably different characteristics.

## 3. Experimental

### 3.1. General

^1^H- (500 MHz; 400 MHz) and ^13^C-NMR (125 MHz; 100 MHz) spectra were obtained on a Bruker DRX-500 and DRX-400 Advanc instrument. IR (ATR) spectra were measured on a Perkin-Elmer Spektrometer 800. Melting points were obtained on a Leica Galen melting point apparatus with Wagner-Munz control unit and are uncorrected. Mass spectra were recorded on a Finnigan MAT 95 SQ by FAB-ionisation (fast atom bombardment). TLC analysis was facilitated by the use of KMnO_4_/H_2_O in addition to UV light (254 nm, 366 nm) with fluorescent-indicating plates (silica gel, Merck, 60 F 254, layer thickness 0.2 mm). The solvents were purchased in absolute quality or dried as follows: THF was dried over sodium/benzophenone, DCM and *n*-hexane over CaH_2_. RCM reactions were conducted in Schlenk-type glassware or in a Carousel Reaction Station (Radleys Discovery Technologies Company). If needed, substrates have been distilled prior to use. 

### 3.2. General Procedure for the Synthesis of Propargyl Alcohols ***8a***–***c***

A dry and nitrogen-flushed 25 mL Schlenk-flask equipped with a magnetic stirring bar and septum was charged with the benzophenone-derivative **10** (1 equiv.), which was dissolved in dry THF (c = 2.2 M). The solution was cooled to 0 °C followed by dropwise addition of ethynylmagnesium bromide (1 equiv., solution in THF 0.5 M) within 15 min. After 1 h the solution was allowed to reach RT and was stirred until full conversion was detected by TLC control. The solution was quenched by adding aqueous saturated NH_4_Cl and subsequently diluted with Et_2_O. After separation of the two layers the aqueous phase was extracted with Et_2_O (3×). The combined organic phases were washed with brine and dried over MgSO_4_. Filtration and subsequent evaporation afforded a crude mixture, which was purified by flash chromatography to yield the target compound. The spectroscopic data obtained for **8c [28]** were consistent with those reported in the literature.

*1,1-bis-(4-Methylphenyl)prop-2-yn-1-ol* (**8a**): Compound **10a** (0.5 g, 2.4 mmol) and ethynyl-magnesium bromide (4.8 mL, solution in THF 0.5 M, 2.4 mmol) were reacted following the general procedure. Chromatographic purification [*n*-hexane/EE 7:1 (v:v)]; R_f_ = 0.26) provided 0.4 g of **8a** (1.7 mmol; 70%) as yellow solid. ^1^H-NMR (CDCl_3_, 400 MHz): δ 2.35 (s, 6H), 2.72 (s, 1H), 2.87 (s, 1H), 7.80 (d, *J* = 8.5 Hz, 4H), 7.50 (d, *J* = 8.5 Hz, 4H); ^13^C-NMR (CDCl_3_, 100 MHz): δ 21.0, 74.2, 75.3, 86.7, 126.0, 129.2, 137.8, 142.0; IR (ATR): 3539, 3450, 3286, 2923, 1717, 1510, 821 cm^−1^; EI-MS (60 °C): *m/z* (%) 236 (100) [M^+^], 221 (84), 145 (57), 91 (57), 53 (60); HR-MS: (M^+^ = C_17_H_16_O) found: 236.1208, calc.: 236.1201.

*1,1-bis-(4-Nitrophenyl)prop-2-yn-1-ol* (**8b**): Compound **10b** (0.8 g, 2.9 mmol) and ethynylmagnesium bromide (5.9 mL, solution in THF 0.5 M, 2.9 mmol) were reacted following the general procedure. Chromatographic purification [*n*-hexane/EE 3:1 (v:v)]; R_f_ = 0.29) provided 0.7 g of **8b** (2.3 mmol; 78%) as yellow solid. ^1^H-NMR (CD_2_Cl_2_, 400 MHz): δ 3.05 (s, 1H), 3.07 (s, 1H), 7.80 (d, *J* = 9.2 Hz, 4H), 8.21 (d, *J* = 8.8 Hz, 4H); ^13^C-NMR (CD_2_Cl_2_, 100 MHz): δ 73.3, 77.9, 83.9, 123.9, 126.9, 147.7, 149.8; IR (ATR): 3486, 3288, 2927, 2588, 2117, 1700, 1608, 1519, 1345, 702 cm^−1^; EI-MS (200 °C): *m/z* (%) 298 (14) [M]+, 281 (43), 251 (52), 176 (100); HR-MS (m/e) found for (M^+^) 298.0588, calc. for C_15_H_10_N_2_O_5_ 298.0590.

### 3.3. General Procedure for the Synthesis of the Precatalysts ***5a***–***c***

A dry and nitrogen-flushed Schlenk-tube was successively charged with RuCl_2_ (PPh_3_)_3_ (1.00 equiv.), **X** (1.15 equiv.), PCy_3_ (2.30 equiv.) and dry THF (c = 0.025 M). The solution was heated to 70 °C for 16 h, whereupon the solvent was removed to afford the crude mixture. Dissolved in a minimum amount of DCM the residue was treated with an excess of *n*-hexane to obtain a precipitate, which was filtered and washed with cold *n*-hexane to yield the desired precatalyst.

*(PCy_3_)_2_Cl_2_Ru[bis-(4-methylphenyl)-allenylidene]* (**5a**): The reaction of RuCl_2_(PPh_3_)_3_ (100 mg, 0.10 mmol), **8a** (28.3 mg, 0.12 mmol), PCy_3_ (67.3 mg, 0.24 mmol) afforded **5a** (94.9 mg, 0.10 mmol, 96%) as an orange brown powder after precipitation, following the general procedure. ^1^H-NMR (CD_2_Cl_2_, 500 MHz): δ 1.13–1.26 (m, 24H), 1.42–1.49 (m, 12H), 1.65–1.67 (m, 12H), 1.98 (d, *J* = 12.2 Hz, 12H), 2.18 (s, 6H), 2.60–2.65 (m, 6H), 7.10 (d, *J* = 8.0 Hz, 4H), 7.70 (d, *J* = 8.2 Hz, 4H); ^13^C-NMR (CD_2_Cl_2_, 125 MHz): δ 21.5, 26.6, 28.0, 29.7, 32.1, 129.5, 129.8, 139.0, 143.9, 144.3, 186.3, 214.2; ^31^P-NMR (CD_2_Cl_2_, 125 MHz): δ 40.6; IR (ATR): cm^−1^ 3411, 2924, 2849, 1913, 1715, 1436; FAB-MS: *m/z* (%) 951 (40) [M]^+^, 878 (100) [M−2Cl]^+^.

*(PCy_3_)_2_Cl_2_Ru[bis-(4-nitrophenyl)-allenylidene]* (**5b**): The reaction of RuCl_2_(PPh_3_)_3_ (100 mg, 0.10 mmol), **8b** (35.8 mg, 0.12 mmol), PCy_3_ (67.3 mg, 0.24 mmol) afforded **5b** (57.3 mg, 0.06 mmol, 57%) as a dark brown powder after precipitation, following the general procedure. ^1^H-NMR (CD_2_Cl_2_, 500 MHz): δ 1.12–1.25 (m, 24H), 1.41–1.48 (m, 12H), 1.66 (d, *J* = 10.7 Hz, 12H), 1.92 (d, *J* = 11.9 Hz, 12H), 2.55–2.65 (m, 6H), 7.95 (d, *J* = 8.7 Hz, 4H), 8.18 (d, *J* = 9.1 Hz, 4H); ^13^C-NMR (CD_2_Cl_2_, 125 MHz): δ 26.4, 27.1, 29.5, 32.4, 114.9, 131.7, 140.5, 146.2, 160.4, 190.1, 216.8; ^31^P-NMR (CD_2_Cl_2_, 125 MHz): δ 44.4; IR (ATR): cm^−1^ 3348, 3076, 2929, 2854, 1926, 1525, 1347, 696; FAB-MS: *m/z* (%) 940 (39) [M−2Cl]^+^, 843 (100), 819 (60).

*(PCy_3_)_2_Cl_2_Ru[bis-(4-methoxyphenyl)-allenylidene]* (**5c**): The reaction of RuCl_2_(PPh_3_)_3_ (200 mg, 0.21 mmol), **8c** (64.4 mg, 0.24 mmol), PCy_3_ (134 mg, 0.48 mmol) afforded **5c** (155 mg, 0.16 mmol, 76%) as a reddish brown powder after precipitation, following the general procedure. ^1^H-NMR (CD_2_Cl_2_, 500 MHz): δ 1.13–1.24 (m, 24H), 1.42–1.50 (m, 12H), 1.65–1.67 (m, 12H), 1.99 (d, *J* = 12.4 Hz, 12H), 2.37 (s, 3H), 2.57–2.69 (m, 6H), 6.76 (d, *J* = 8.8 Hz, 2H), 7.23 (t, *J* = 8.1 Hz, 2H), 7.45 (t, *J* = 7.1 Hz, 1H), 7.67 (d, *J* = 7.5 Hz, 2H), 7.75 (d, *J* = 9.6 Hz, 2H); ^13^C-NMR (CD_2_Cl_2_, 125 MHz): δ 26.6, 28.0, 29.7, 32.1, 55.5, 114.1, 131.3, 139.9, 160.3, 188.6, 215.5; ^31^P-NMR (CD_2_Cl_2_, 125 MHz): δ 40.1; IR (ATR): cm^−1^ 3368, 3056, 2928, 2846, 1932, 1595, 1252, 1169, 695; FAB-MS: *m/z* (%) 983 (28) [M]^+^, 910 (100) [M−2Cl]^+^, 797 (30), 613 (60), 531 (46).

## 4. Conclusions

In this communication we reported on the first synthesis of electronically modified neutral 16 VE Ru-based allenylidene precatalysts. With the aim of modifying the carbene moiety in **5** (Figure 1) at the *para*-positions within the aromatic systems, the classical dehydration approach rendered a flexible access for the intended alteration. With regard to this, substituted propargyl alcohols **8a**–**c** were derived in good isolated yields (63–87%) by the addition of ethynylmagnesium bromide to the respective benzophenone derivatives **10a**–**c**. The subsequent dehydration in the presence of **9** and PCy_3_ provided electronically modified allenylidenes **5a**–**c** in moderate to excellent isolated yields (54–96%) after precipitation. Compounds **8a**–**c** were evaluated in RCM of benchmark substrates **6** and **7**; these investigations demonstrated the dramatic differences concerning the initiation rate and stability during metathesis reactions comparing the Me-substituted allenylidene **8a** to the NO_2_-substituted **8b** and OMe-substituted complexes **8c**. 
